# Clinical Assessment of Medical Device–Related Pressure Injury Risk: Profiling Risk Levels in Patients Using Medical Devices

**DOI:** 10.3390/healthcare14131942

**Published:** 2026-07-01

**Authors:** Handan Aydin Kahraman, Gülay İpek Çoban, Ebru Bozcu Kartal

**Affiliations:** 1 Department of Nursing, Faculty of Health Sciences, Erzincan Binali Yıldırım University, Erzincan 24100, Turkey; 2 Department of Nursing, Faculty of Nursing, Ataturk University, Erzurum 25240, Turkey; gulaycoban@atauni.edu.tr; 3 Institute of Health Sciences, Ataturk University, Erzurum 25240, Turkey; ebru.bozcu@erzincan.edu.tr

**Keywords:** critical care, patient safety, medical device-related pressure injury, medical device-related pressure injury scale, pressure injury, nursing assessment, risk assessment

## Abstract

Objective: This study aimed to evaluate the risk of medical device-related pressure injury (MDRPI) development among patients exposed to medical devices and to assess the clinical utility of the Medical Device-Related Pressure Injury Risk Assessment Scale (MDRPIS). Methods: This clinical assessment study included 132 patients receiving care in intensive care, palliative care, and home-care units. The MDRPIS total score ranges from 8 to 27, with scores of 8–12 indicating high risk, 13–21 indicating moderate risk, and 22–27 indicating low risk. The scale was used to assess MDRPI risk associated with life-sustaining medical devices. Its psychometric performance was evaluated through analyses of internal consistency, criterion validity against the Braden Scale, and diagnostic accuracy using receiver operating characteristic (ROC) analysis. Results: The MDRPIS demonstrated strong discriminative ability for identifying patients at risk of MDRPI, with an area under the curve (AUC) of 0.822. A cut-off score of ≤16 was identified as the optimal threshold for detecting high-risk patients. Patients with MDRPIS scores of 16 or lower had a significantly higher incidence of MDRPI than those classified as low risk (*p* < 0.001). Respiratory support devices, particularly non-invasive ventilation (NIV)/continuous positive airway pressure (CPAP) masks and tracheostomy flanges or securement devices, were identified as the most significant risk factors for injury development. The highest incidence of MDRPI was observed among patients in intensive care units, followed by those in palliative care and home-care settings, indicating a statistically significant concentration of device-related risk in high-acuity care environments (*p* < 0.05). Conclusions: Clinical settings, particularly intensive care and palliative care units, should incorporate the MDRPIS into routine risk assessment protocols to facilitate targeted preventive interventions, including prophylactic dressings and advanced fixation techniques for patients using high-risk devices such as NIV masks and tracheostomy securement systems. The systematic implementation of the MDRPIS may support more effective allocation of nursing resources and enhance patient safety by enabling the early identification and prevention of avoidable device-related pressure injuries. Furthermore, the findings indicate that an MDRPIS score of 16 or below represents a clinically meaningful threshold for initiating preventive interventions.

## 1. Introduction

Pressure injury (PI) remains a persistent and widespread problem in healthcare systems worldwide, posing a substantial threat to patient safety. It typically develops in individuals who are confined to a bed or chair for extended periods and is caused by sustained pressure on the skin and underlying tissues [1]. With the increasing integration of technology into clinical care processes, the etiology of PI has expanded beyond the traditional concept of “bedsores,” and medical device-related pressure injuries (MDRPIs) have emerged as a distinct and clinically significant subgroup [2]. Reflecting this evolution, the National Pressure Injury Advisory Panel (NPIAP) revised the definition of PI to emphasize the detrimental effects of medical devices on soft tissues and mucosal membranes. Furthermore, the definition of MDRPI was broadened to encompass injuries associated with non-medical devices, such as bed frames and household items. The NPIAP also recommended that MDRPIs be assessed and documented independently from conventional pressure injuries [3,4]. Critically ill patients admitted to intensive care units (ICUs), who are frequently exposed to multiple medical devices and prolonged hospitalization, constitute the population at greatest risk for MDRPI regardless of age. Black and Cuddigan reported that the incidence of MDRPI among critically ill patients ranges from 0.9% to 41.2% [5], highlighting substantial variability across high-acuity care settings. Moreover, exposure to multiple medical devices has been associated with a 1.16- to 2.4-fold increase in the risk of MDRPI development among critically ill patients [6,7]. A fundamental distinction between MDRPI and traditional pressure injuries is that MDRPI may develop not only over bony prominences but also in any anatomical area where a medical device comes into contact with the skin or mucosal tissue. In addition, tissue damage may occur considerably more rapidly than in conventional pressure injuries [8]. Medical devices that exert localized pressure, particularly respiratory support devices such as non-invasive ventilation (NIV) masks and endotracheal tubes, as well as catheters and fixation materials, contribute to tissue ischemia through mechanical loading, friction, shear forces, and microclimate alterations at the skin–device interface [9,10]. Recent clinical studies have demonstrated that MDRPI, as a distinct patient safety concern, has become an increasingly prevalent problem across healthcare settings worldwide [9]. Failure to identify MDRPI at an early stage not only complicates wound management but may also result in preventable adverse events being perceived as hospital-acquired complications and potential indicators of inadequate care quality [11]. Consequently, international guidelines classify all patients using medical devices as being at elevated risk for MDRPI and emphasize the implementation of structured nursing interventions, including routine assessment of device contact areas, early detection of tissue damage, application of prophylactic dressings, and pressure redistribution strategies [12,13,14]. Existing traditional risk assessment scales, such as the Braden or Norton scales, were never originally designed to assess for MDRPIs, as they primarily focus on systemic patient immobility. These instruments primarily focus on patient-related factors like immobility and physiological status, while failing to account for extrinsic mechanical pressure and the cumulative effect of multiple device exposures [15,16]. To address this critical gap in the literature, Kahraman and Çoban (2025) recently developed and validated the Medical Device-Related Pressure Injury Risk Assessment Scale (MDRPIS), providing a psychometrically robust and device-specific framework for clinicians [17]. While the development of the MDRPIS marked a pivotal shift toward objective risk management, its clinical utility and diagnostic performance in diverse acute and intensive care settings require further empirical validation. Consequently, this study was designed to implement the MDRPIS within a clinical population to evaluate its effectiveness in identifying high-risk patients. By transitioning from the initial development phase to systematic clinical application, this research aims to standardize MDRPI risk assessment, ultimately bridging the gap between evidence-based nursing practice in complex clinical environments.

## 2. Materials and Methods

### 2.1. Study Design and Participants

This study was conducted to evaluate the risk of MDRPI. The study was conducted in the adult intensive care units and palliative care clinics of a public hospital located in eastern Turkey. Data collection was conducted between January and March 2024 following the receipt of institutional approval.

Inclusion Criteria:

- Patients aged >18 years;
- Hospitalized for at least 24 h;
- Having at least one medical device (e.g., endotracheal tube, nasogastric tube, urinary catheter) in contact with the skin or mucosa;
- Having no pre-existing pressure injuries upon admission.

To precisely clarify the cross-sectional study framework, the methodology is structured upon three core operational elements: (1) an objective non-interventional observation design, (2) a dynamic single-center clinical validation framework, and (3) a patient-centered baseline assessment strategy. Participant recruitment was conducted continuously during routine intensive and palliative care admission workflows, ensuring that patients were screened immediately upon meeting all inclusion criteria within their first 24 h of exposure to any life-sustaining equipment.

### 2.2. Ethical Considerations

The study was conducted in accordance with the Declaration of Helsinki. Prior to the commencement of the study, ethical approval was obtained from the Erzincan Binali Yıldırım University Human Research Health and Sport Sciences Ethics Committee (Date: 27 October 2023, Protocol No: 10/05). Following the ethical approval, official institutional permission was obtained from the administration of the public hospital where the study was conducted. Participation was strictly voluntary, and all participants or their legal representatives were informed about the study’s purpose and procedures. Written informed consent was obtained from all participants or their legal guardians before data collection, ensuring that their anonymity and confidentiality were maintained.

### 2.3. Data Collection and Instruments

Data were collected through direct clinical observation and patient record reviews using the following instruments:

Patient Information Form: Captured demographic data (age, gender) and clinical characteristics (diagnosis, type of medical device).

The Braden Scale: The Braden Scale was used as the reference standard to evaluate the general pressure injury risk and to establish the concurrent validity of the MDRPIS. Developed by Bergstrom et al. (1987) [18], the scale consists of six subscales: sensory perception, moisture, activity, mobility, nutrition, and friction/shear. Total scores range from 6 to 23, with lower scores indicating a higher risk of pressure injury development. The Braden Scale is a globally recognized tool with well-established validity and reliability across various clinical settings, including intensive care and long-term care units. The Braden Scale has demonstrated strong psychometric properties in various clinical populations, with reported Cronbach’s alpha coefficients ranging from 0.70 to 0.89, indicating high internal consistency [18,19]. Additionally, its inter-rater reliability has been consistently high (ICC > 0.80), making it a reliable ‘gold standard’ for evaluating the concurrent validity of new assessment tools.

The Medical Device-Related Pressure Injury Risk Assessment Scale (MDRPIS): The MDRPIS was developed by Kahraman and Çoban (2025) [17] specifically to address the limitations of general pressure injury scales in predicting risks associated with medical devices. The scale consists of 8 items evaluating clinical and device-related factors: (1) General risk (based on Braden), (2) Consciousness level, (3) Tissue perfusion, (4) Skin/mucosa integrity at the contact site, (5) Friction, (6) Device placement (under the patient’s body), (7) Use of pressure-relieving materials, and (8) Duration of device exposure.

Items are scored on a Likert-type scale ranging from 1 to 3 or 4 points. The total score ranges from 8 to 27, where lower scores indicate a higher risk of MDRPI development. In the original development study, the scale was reported to have high validity and reliability.

The scale operates on an inverse scoring logic, where lower total scores indicate a progressively higher risk of MDRPI development. The total score range (8–27) is categorized into three clinical risk levels:

High Risk (8–12 points): Indicates a critical need for immediate intervention and advanced pressure-relieving strategies.

Moderate Risk (13–21 points): Requires close monitoring and supportive skin protection measures.

Low Risk (22–27 points): Routine clinical observations are considered sufficient.

The scale integrates both physiological and device-specific parameters to provide a comprehensive risk profile. Key physiological factors include tissue perfusion (e.g., sPO_2_ < 70%, Blood Pressure < 60 mmHg) and consciousness levels, which are critical as impaired oxygenation and sensory perception accelerate tissue breakdown under mechanical pressure. Furthermore, device-related factors such as friction intensity, device placement (e.g., remaining under the patient’s body for >4 h), and duration of exposure are heavily weighted to reflect the mechanical etiology of MDRPIs. For concurrent validity, the Braden Scale is integrated as a reference, where a Braden score of 12 is cross-referenced with high-risk status in the MDRPIS [17]. For the present study population (N = 132), the internal consistency of the MDRPIS was evaluated, and the Cronbach’s Alpha coefficient was found to be 0.646. This value is considered acceptable for a clinical assessment tool focusing on multi-dimensional clinical risk factors in acute and intensive care settings.

It is important to emphasize that the current study does not intend to re-evaluate the baseline structural framework of the MDRPIS through Exploratory (EFA) or Confirmatory Factor Analysis (CFA), as its psychometric structure and construct validity were already rigorously established and validated during the original scale development phase by Kahraman and Çoban (2025) [17]. Instead, this research serves as the first independent clinical application and diagnostic accuracy validation study of the tool within an acute and critical care patient cohort.

Regarding the scale parameters, consciousness level in the MDRPIS explicitly evaluates patient sedation depth and potential for self-device manipulation, which clinically differs from the sensory perception subscale of the Braden tool, thus avoiding statistical double-counting. Friction intensity at the patient-device interface was monitored based on clinical observation of device micro-movements caused by patient repositioning, ventilator circuit tension, or patient agitation, scored on the instrument’s standardized clinical response guidelines.

### 2.4. Procedure

The researchers, who are experienced in wound care and intensive care nursing, performed systematic daily assessments. Each participant was evaluated using both the Braden Scale and the MDRPIS simultaneously. MDRPI presence and staging were determined based on the NPIAP/EPUAP international guidelines. To ensure consistency, assessments were performed at the same time each day during routine nursing care rounds.

### 2.5. Data Analysis

Statistical analyses were performed using SPSS version 27.0 (IBM Corp., Armonk, NY, USA). The normality of the data was tested using the Shapiro–Wilk test. Since the distribution was non-normal (*p* < 0.05), non-parametric tests were employed. Associations between categorical variables were analyzed using Pearson’s chi-square test or Fisher’s exact test, as appropriate. Descriptive Statistics: Frequencies, percentages, and means standard deviations were calculated. Validity and Reliability: Internal consistency was evaluated using Cronbach’s alpha. The Mann–Whitney U test was used to compare MDRPIS scores between groups with and without injuries. Diagnostic Performance: Receiver Operating Characteristic (ROC) curve analysis was conducted to determine the Area Under the Curve (AUC), optimal cut-off score, sensitivity, specificity, and predictive values. The significance level was set at *p* < 0.05.

To ensure statistical independence and avoid unit-of-analysis errors associated with multi-device exposure on a single patient, the primary patient-level analysis was strictly maintained. For patients exposed to multiple concurrent medical devices, the analysis uniquely focused on the single primary device identified by the clinical investigators as exerting the highest localized mechanical pressure or being directly associated with the primary anatomical site of pressure injury development. Consequently, each patient contributed exactly one primary device lineage to the contingency matrices, preventing statistical dependencies or inflated cell counts. For patients exposed to multiple concurrent medical devices, the analysis uniquely focused on the single primary device identified by the clinical investigators as exerting the highest localized mechanical pressure or being directly associated with the primary anatomical site of pressure injury development. Consequently, each participant contributed exactly one primary device lineage to the cross-tabulation matrices, avoiding nested data dependencies or inflated cell counts in the Chi-square and Fisher’s exact models.

## 3. Results

### 3.1. Demographic and Clinical Characteristics of Participants

A total of 132 participants completed the study. The mean age of the participants was 65.80 ± 16.94 years, and 53.8% (n = 71) were male. Most participants were hospitalized in intensive care units (48.5%, n = 64), followed by palliative care clinics (21.2%, n = 28), home-care units (15.2%, n = 20), surgical clinics (9.1%, n = 12), and internal clinics (6.0%, n = 8). The most commonly used medical devices were respiratory masks (NIV/CPAP) (22.7%), tracheostomy flanges/securement lines (19.7%), endotracheal tubes (18.2%), and nasogastric tubes (13.6%) (Table 1).

While Table 1 provides a comprehensive overview of the patients’ demographic and clinical profiles, it also highlights the variety of medical devices used during their treatment.

### 3.2. Device Type and Injury Development

To further understand the clinical impact of these exposures, the relationship between specific device types and the development of pressure injuries was analyzed. Chi-square or Fisher’s exact test was used depending on expected cell counts. (Chi-square/Fisher’s exact test, *p* < 0.05). Patients using respiratory masks (NIV/CPAP) and tracheostomy flanges/securement lines had the highest frequency of injury development. Significantly higher injury rates were observed among patients using respiratory masks and nasogastric tubes compared with patients without injury.

**Figure 1:**
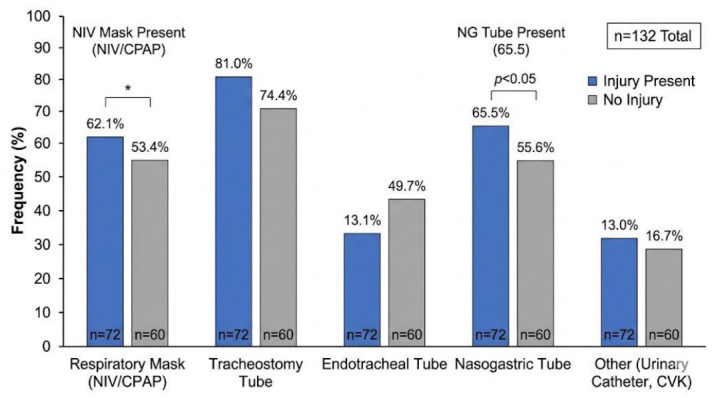
Distribution of primary medical device exposure types by pressure injury presence (n = 132). *: Statistically significant at the 0.05 level.

The relationship between medical device types and the development of pressure injuries was analyzed to determine device-specific risk profiles. As presented in Table 2, the incidence of injuries varied significantly depending on the device used. Specifically, patients using respiratory masks, tracheostomy flanges/securement lines, and nasogastric tubes demonstrated a higher propensity for injury development. To further visualize these clinical findings and highlight the devices with the highest risk frequency, the distribution of MDRPI according to device type is illustrated in Figure 1.

While this categorical distribution displays the absolute frequency of primary device exposure interfaces, the cumulative temporal dimension specifically the ‘Duration of Device Exposure’ is mathematically accounted for and structured within the multidimensional MDRPIS total risk index (Item 8), rather than being visually continuous on this bivariate graph outline. (Note: Tracheostomy tube injuries are predominantly localized around the tube flange interface.).

Analysis revealed that the highest incidence of MDRPI occurred in patients using Respiratory Masks (NIV/CPAP) and Tracheostomy flanges/securement lines, accounting for the majority of observed injuries. Specifically, patients with pressure injuries showed significantly higher exposure rates to non-invasive ventilation masks compared to the non-injury group (*p* < 0.05). Additionally, significant injury development was observed in patients with Nasogastric Tubes, whereas other devices such as urinary catheters and central venous catheters (CVC) were associated with a lower frequency of injury development.

### 3.3. Reliability and Construct Validity of the MDRPIS

Table 3 presents the psychometric evaluation of the MDRPIS, detailing its internal consistency, criterion validity in relation to the Braden Scale, and its effectiveness in discriminating risk between patient groups.

The internal consistency of the 8-item MDRPIS exhibited acceptable internal consistency with a Cronbach’s alpha of 0.646. Considering the multidimensional structure of the scale and the limited number of items, this value may be considered acceptable for a newly developed clinical screening tool. Criterion validity was strongly supported by a significant positive correlation with the Braden Scale (r = 0.840, *p* < 0.001).

**Figure 2:**
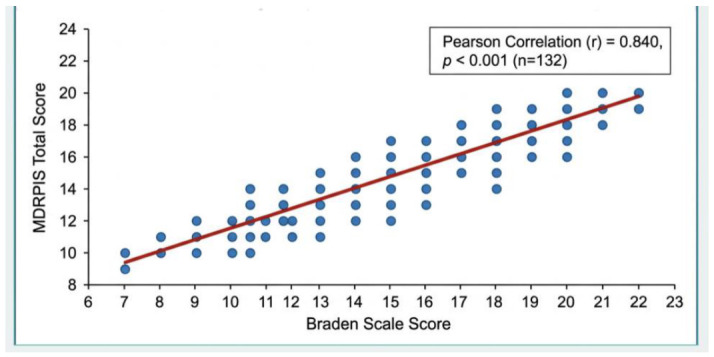
Scatter plot illustrating the linear relationship and strong positive correlation between the Braden Scale scores and the Medical Device-Related Pressure Injury Risk Assessment Scale (MDRPIS) total scores (r=0.840,p<0.001,n=132). Note: The solid red line represents the linear regression line indicating a strong positive correlation between the Braden Scale and MDRPIS scores.

The individual contribution of each scale item to the development of MDRPI is detailed in Table 3, showing that specific factors like device exposure and friction are significantly associated with injury status. To visualize the collective impact of these items, the distribution of total MDRPIS scores is presented in Figure 2, which clearly illustrates the significant separation between patients with and without injuries based on their overall risk profiles.

**Figure 3:**
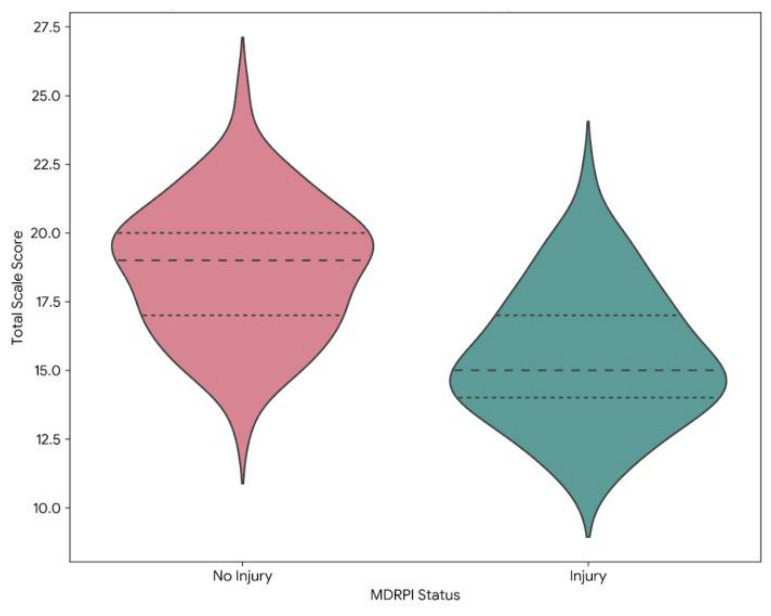
Violin plot showing the distribution and density of the Medical Device-Related Pressure Injury Scale (MDRPIS) total scores. Note: The wider sections of the violin represent a higher frequency of patient scores, while the internal dashed lines indicate the median and interquartile ranges (IQR).

To further evaluate the discriminant validity of the MDRPIS, the total scores were compared between patients with and without medical device-related pressure injuries. A highly significant difference was observed between the two groups (*p* < 0.001). The mean total score for patients who developed MDRPI was significantly lower (15.49 ± 2.41) compared to those who did not develop an injury (18.62 ± 2.39). This definitive score distribution and the significant separation between the groups are visually demonstrated in the violin plot. This difference was statistically significant (*p* < 0.001). The score distribution across groups is visualized in the violin plot (Figure 3).

Distribution of total MDRPIS scores according to the presence of pressure injuries (n = 132). The violin plot demonstrates a significant shift towards lower scores (indicating higher risk) in the injury-positive group compared to the no-injury group (Figure 3).

### 3.4. Diagnostic Performance and Predictive Validity

Based on Youden’s index, the optimal cut-off score was 16. At this threshold, the scale demonstrated a sensitivity of 67.6% and a specificity of 80.0%. The positive predictive value (PPV) was 80.0%, and the negative predictive value (NPV) was 67.6%. Detailed diagnostic performance metrics are summarized in Table 4.

**Figure 4:**
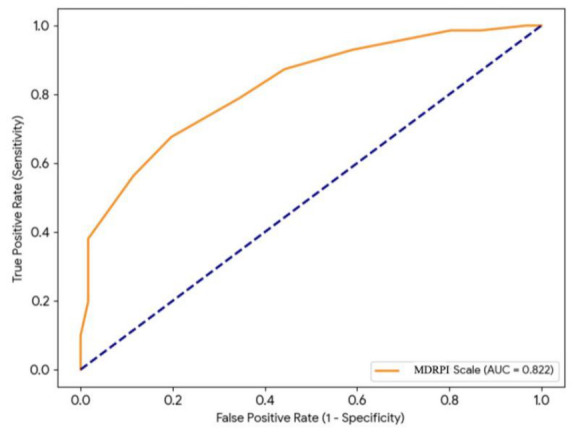
Receiver Operating Characteristic (ROC) curve demonstrating the diagnostic accuracy and predictive validity of the Medical Device-Related Pressure Injury Risk Assessment Scale (MDRPIS) (n = 132), with an Area Under the Curve (AUC) of 0.822 (95% CI: 0.751–0.893). Note: The diagonal dashed line represents the reference line of chance (AUC = 0.50).

The diagnostic accuracy of the MDRPIS was evaluated using the Characteristic (ROC) curve analysis, as shown in Figure 4. This AUC value confirms the scale’s capability in predicting MDRPI, with an optimal cut-off score of 16 providing a balance between sensitivity and specificity.

The predictive ability of the MDRPIS in identifying patients at risk for MDRPI was evaluated using ROC curve analysis (Figure 4). The Area Under the Curve (AUC) was determined to be 0.822 (95% CI: [0.751–0.893]), representing good diagnostic accuracy.

### 3.5. Item-Based Risk Distribution

**Figure 5:**
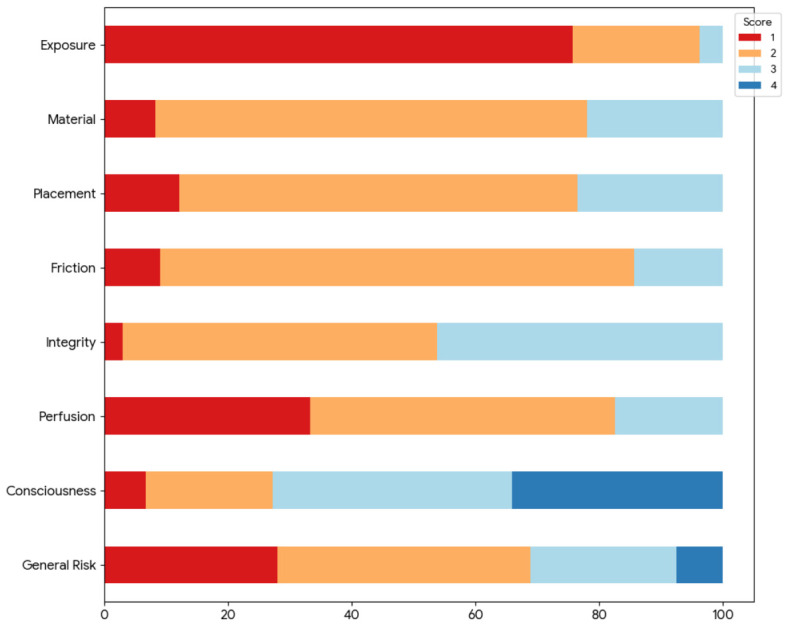
Item-based risk distribution and proportional percentages for the Medical Device-Related Pressure Injury Scale (MDRPIS) (n=132). The color-coded bars represent the distribution of patient scores for each of the eight scale items, ranging from Score 1 (High Risk, highlighted in red/darker colors) to Score 4 (No Risk, highlighted in blue/lighter colors), allowing for a comprehensive visual interpretation of item-specific risk severity.

Figure 5 Risk distribution across the eight items of the MDRPIS for the 132 study patients. Scores represent risk levels (1: High Risk/Red, 4: No Risk/Blue), visually highlighting critical clinical factors that contribute to medical device-related pressure injury development. The proportional distribution of risk levels across all eight individual items of the scale for the study population is presented in Figure 5.

Detailed item-based analysis reveals that mechanical factors such as device exposure duration and friction contributed most significantly to the high-risk categorization of the participants. Each bar represents the percentage of patients categorized into risk levels from 1 (High Risk, shown in red) to 4 (No Risk, shown in blue/green). An analysis of individual scale items revealed that “Device Exposure Duration,” “Friction,” and “Device Placement” were the factors most frequently associated with high-risk scores among the participants. The proportional distribution of risk levels across all eight items is shown in Figure 5.

## 4. Discussion

This study was conducted to determine the risk levels of MDRPIs in healthcare settings and to evaluate the diagnostic performance of the newly developed MDRPIS. The findings indicate that patients, particularly those treated in intensive care units (ICUs), are at considerably higher risk, and that specific medical devices are directly associated with the development of pressure injuries.

In the present study, MDRPI was developed in 54.5% (n = 72) of the participants. Previous studies have reported MDRPI incidence rates in ICU patients ranging from 5% to 31% [20]. The higher rate observed in our study may be attributed to the fact that 48.5% of our sample consisted of ICU patients and to the frequent use of high-risk devices such as NIV masks and tracheostomy flanges/securement lines. Indeed, NIV/CPAP masks (22.7%) and tracheostomy flanges/securement lines (19.7%) were identified as the most commonly used devices and those most significantly associated with injury development (*p* < 0.05). This finding is consistent with studies reporting that NIV masks account for up to 52.6% of MDRPI cases. Specifically, clinical evidence revealed that tissue breakdown associated with tracheostomy support predominantly developed around the tracheostomy tube flanges and securement lines, where rigid plastic material exerts localized mechanical tension on the neck anatomy [21]. Furthermore, while the physical stiffness of the device plastic represents a profound biomechanical risk factor, it was not directly measured in this observational phase, highlighting an avenue for future engineering-nursing collaborative research.

Device-specific risk analysis further demonstrated that respiratory masks and nasogastric (NG) tubes play a critical role in the development of pressure injuries (*p* < 0.05). Jo and Choi (2023) similarly emphasized that NG tubes are among the most common causes of MDRPIs in ICU settings [7]. In our study, “device exposure duration” and “friction” were the most frequently observed factors in the high-risk category, supporting the importance of mechanical load and prolonged skin-device contact in the pathogenesis of these injuries. Consistent with this, the literature indicates that each additional day of hospitalization increases the likelihood of MDRPI development [22].

The psychometric evaluation of the MDRPIS demonstrated that the scale is a valid and reliable tool for clinical use. The MDRPIS achieved a Cronbach’s alpha of 0.646, which is considered acceptable and sufficient for a newly developed clinical tool consisting of eight diverse clinical parameters. Given that the scale integrates distinct physiological and mechanical factors, the marked difference in mean scores indicates that the scale effectively distinguishes between different risk levels, confirming its clinical utility in identifying patients at high risk for injury development. The Cronbach’s alpha coefficient (0.646) is considered acceptable for a newly developed, multidimensional screening instrument [23]. The strong positive correlation with the Braden Scale (r = 0.840, *p* < 0.001) supports its criterion validity. However, while the Braden Scale focuses on general pressure injury risk, the MDRPIS provides a more specific assessment by incorporating device-related factors such as friction, positioning, and exposure duration, thereby offering a distinct clinical advantage. Unlike traditional pressure injuries where mobility is the leading concern, MDRPI risk is predominantly dictated by the physical presence and duration of medical equipment. This finding emphasizes that nursing interventions should prioritize frequent site rotations and skin inspections for devices that remain in contact for more than 2 h, as highlighted by the high frequency of ‘high-risk’ scores in these categories.

The area under the curve (AUC) value of 0.822 indicates good diagnostic accuracy. In clinical validation studies, an AUC between 0.80 and 0.90 is classified as “very good” discriminative power. This result proves that the MDRPIS can highly accurately distinguish between patients who will develop medical device-related pressure injuries and those who will not. At the ≤16 threshold, the scale achieved a balance of 67.6% sensitivity and 80.0% specificity. This signifies that patients scoring 16 or below are at significantly higher risk, providing a clear objective threshold for clinicians to initiate preventive interventions. The high specificity rate is particularly important as it minimizes “false alarms”, ensuring that intensive preventive resources are directed toward the truly high-risk patients. This value is comparable to that of the MDRPI tool (AUC = 0.861) reported in the literature, suggesting an excellent level of discrimination [16]. Additionally, the significantly lower mean score observed in patients who developed injuries (15.49 ± 2.41; *p* < 0.001) further supports the discriminative validity of the scale. The 95% Confidence Interval (CI: 0.751–0.893) does not include 0.5, confirming that the scale’s predictive ability is statistically significant and not due to chance.

In conclusion, this study demonstrates that the risk profile of patients using medical devices varies depending on device type and duration of exposure. The MDRPIS is a practical, valid, and clinically applicable tool that enables early identification of device-related risks by nurses and clinicians.

### Limitations of the Study

Several methodological limitations must be acknowledged. First, the high incidence of MDRPI (54.5%) observed in this cohort may reflect a potential selection bias, as a substantial proportion of the participants (48.5%) were recruited from high-acuity Intensive Care Units where multi-device exposure is absolute. Second, the single-center cross-sectional design conducted in a public hospital in Eastern Turkey limits the immediate generalizability of the findings to diverse regional or international healthcare structures. Additionally, due to the non-parametric distribution of the clinical dataset, advanced multivariable logistic regression to control for systemic confounders (such as age or unit type) and the DeLong test for direct geometric AUC comparison between the Braden Scale and the MDRPIS were not performed. These advanced modeling protocols remain a clear methodological limitation of the current validation stage. Furthermore, because the optimal clinical cut-off score of ≤16 was derived and evaluated within the same patient sample, there is an inherent risk of optimism bias and statistical overfitting. Future multi-center, prospective longitudinal studies incorporating external cross-validation cohorts are highly warranted to confirm the broader screening generalizability of the MDRPIS.

## 5. Conclusions

This study demonstrates that MDRPI represents a significant clinical challenge, particularly in high-acuity settings such as intensive care and palliative units. The findings highlight a critical shift in pressure injury etiology; while traditional risk assessment models focus heavily on patient mobility and nutrition, MDRPI risk is predominantly driven by the mechanical presence of life-sustaining equipment, the duration of device exposure, and the localized friction at the device-skin interface.

The newly developed MDRPIS tool proved to be a statistically robust and clinically practical instrument. With an AUC of 0.822, the scale exhibits good discriminative power, effectively identifying patients at risk with a clear objective threshold (≤16 points). Despite the diverse nature of the eight clinical parameters, the scale’s internal consistency and strong correlation with the Braden Scale (r = 0.840) confirm its validity as a specialized adjunct to traditional assessment tools.

Crucially, the study identifies respiratory support devices (NIV/CPAP masks and tracheostomy flanges/securement lines) and nasogastric tubes as the highest-risk factors for mucosal and skin integrity compromise. Clinical protocols should therefore prioritize:

Targeted Monitoring: Implementation of the MDRPIS for all patients using high-risk respiratory devices to identify those scoring 16 or below.

Preventive Interventions: Frequent site rotations (every 2 h) and the use of prophylactic dressings at the highest-risk interfaces identified by the scale.

Structured Documentation: Integration of device-specific risk parameters into electronic health records to facilitate early nursing interventions.

In conclusion, the MDRPIS offers an evidence-based approach to profiling patient risk levels, allowing for more efficient allocation of preventive resources. Future research should focus on the longitudinal application of this tool across diverse patient populations and its impact on reducing the overall incidence of hospital-acquired MDRPIs.

## Figures and Tables

**Table 1:** Demographic and Clinical Characteristics of the Participants (n = 132).

Demographic Characteristics		
Age	Mean ± SD = 65.80 ± 16.94	
Gender	n	**%**
Female	61	46.2
Male	71	53.8
Clinical Unit	n	**%**
ICU	64	48.5
Palliative Care Clinic	28	21.2
Home-Care Unit	20	15.2
Surgical Clinics	12	9.1
Internal Clinics	8	6.0
Medical Device Type	n	**%**
Respiratory Mask (NIV/CPAP)	30	22.7
Tracheostomy flanges/securement lines	26	19.7
Endotracheal Tube	24	18.2
NG Tube	18	13.6
Peripheral/Central IV Line	12	9.1
Pulse Oximetry Probe	10	7.6
Cervical Collar/Braces	8	6.1
Others (Drainage tubes, etc.)	4	3.0
Total	132	100

Note: n = number of patients; % = percentage; SD = Standard Deviation; Braden Scale scores range from 6 to 23, where lower scores indicate a higher risk of conventional pressure injuries.

**Table 2:** Association Between Device Type and MDRPI Development.

Device Type	Injury Present (n = 72)	No Injury (n = 60)	*p*-Value	Effect Size (Cramer’s V)	Adjusted *p*-Value (Bonferroni)
NIV/CPAP	22	10	0.012 *	0.16	0.096
Tracheostomy Tube	15	9	0.031 *	0.08	0.248
Endotracheal Tube	12	16	0.004 *	0.12	0.032 *
NG Tube	14	4	0.044	0.19	0.352
Others	8	22	0.022	0.30	0.176

Note: NIV = Non-Invasive Ventilation; CPAP = Continuous Positive Airway Pressure; NG = Nasogastric. To control for the inflation of Type I error due to multiple comparisons across device categories, adjusted *p*-values were calculated using the Bonferroni correction (0.05/8). Statistical significance for the adjusted model was set at * *p* < 0.00625. Effect sizes represent Cramer’s V. Others (IV Line, Pulse Oximetry, Cervical Collar, Drainage, Urinary, etc.).

**Table 3:** Internal Consistency and Item-Total Correlation Analysis of the MDRPIS.

MDRPIS Items	Corrected Item-Total Correlation (r)	Cronbach’s Alpha If Item Deleted
Item 1: General Risk (Braden-based)	0.42	612
Item 2: Consciousness Level	0.38	620
Item 3: Tissue Perfusion	0.45	602
Item 4: Skin/Mucosa Integrity at Site	0.41	615
Item 5: Friction Intensity	0.48	595
Item 6: Device Placement Layout	0.39	618
Item 7: Pressure-Relieving Materials	0.35	628
Item 8: Duration of Device Exposure	0.52	584
Overall Scale Statistics	Cronbach’s Alpha = 0.646	No. of Items = 8

Note: MDRPIS = Medical Device-Related Pressure Injury Risk Assessment Scale. Corrected item-total correlations reflect the multidimensional coherence of the clinical risk parameters. A total Cronbach’s alpha of 0.646 is accepted as structurally stable for clinical screening tools targeting highly heterogeneous mechanical and physiological risk boundaries.

**Table 4:** Diagnostic Performance and Predictive Validity of the MDRPIS (n = 132).

Metric	Value (95% CI)
Area Under the Curve (AUC)	0.822 (0.751–0.893)
Optimal Cut-off Score	≤16
Sensitivity (%)	67.6% (95% CI: 55.4–78.2%)
Specificity (%)	80.0% (95% CI: 67.7–89.2%)
Positive Predictive Value (PPV)	80.0% (95% CI: 67.7–89.2%)
Negative Predictive Value (NPV)	67.6% (95% CI: 55.4–78.2%)
Youden Index	0.476

## Data Availability

The data presented in this study are available on request from the corresponding author due to privacy and ethical restrictions.
